# Notch signaling is required for maintaining stem-cell features of neuroprogenitor cells derived from human embryonic stem cells

**DOI:** 10.1186/1471-2202-10-97

**Published:** 2009-08-17

**Authors:** Sun-Mi Woo, Janghwan Kim, Hyo-Won Han, Jung-Il Chae, Mi-Young Son, Sunwha Cho, Hyung-Min Chung, Yong-Mahn Han, Yong-Kook Kang

**Affiliations:** 1 Development and Differentiation Research Center, KRIBB, 111 Gwahangno, Yuseong-gu, Daejeon 305-806, South Korea; 2 Stem Cell Research Laboratory, CHA Stem Cell Institute, Pochon Cha University, Seoul, Republic of Korea; 3 Department of Biological Sciences, Korean Advanced Institute of Science and Technology, Guseong-Dong, Yuseong-Gu, Daejeon 305-701, South Korea

## Abstract

**Background:**

Studies have provided important findings about the roles of Notch signaling in neural development. Unfortunately, however, most of these studies have investigated the neural stem cells (NSCs) of mice or other laboratory animals rather than humans, mainly owing to the difficulties associated with obtaining human brain samples. It prompted us to focus on neuroectodermal spheres (NESs) which are derived from human embryonic stem cell (hESC) and densely inhabited by NSCs. We here investigated the role of Notch signaling with the hESC-derived NESs.

**Results:**

From hESCs, we derived NESs, the *in-vitro *version of brain-derived neurospheres. NES formation was confirmed by increased levels of various NSC marker genes and the emergence of rosette structures in which neuroprogenitors are known to reside. We found that Notch signaling, which maintains stem cell characteristics of *in-vivo*-derived neuroprogenitors, is active in these hESC-derived NESs, similar to their *in-vivo *counterpart. Expression levels of Notch signaling molecules such as NICD, DLLs, JAG1, HES1 and HES5 were increased in the NESs. Inhibition of the Notch signaling by a γ-secretase inhibitor reduced rosette structures, expression levels of NSC marker genes and proliferation potential in the NESs, and, if combined with withdrawal of growth factors, triggered differentiation toward neurons.

**Conclusion:**

Our results indicate that the hESC-derived NESs, which share biochemical features with brain-derived neurospheres, maintain stem cell characteristics mainly through Notch signaling, which suggests that the hESC-derived NESs could be an *in-vitro *model for *in-vivo *neurogenesis.

## Background

Neural stem cells (NSCs), which have properties of self-renewal and differentiation into neurons and glias, are usually isolated from fetal and adult brains in the form of floating clonal derivatives of the NSCs placed in culture, known as neurospheres (NSs) [1-3]. NSCs have the potential to be used in cell replacement therapy for neural disorders such as Parkinson's disease and Alzheimer's disease as well as other neurological disorders including spinal cord injuries [4]. For therapy, maintenance and expansion of the NSCs are necessary to provide sufficient amount of cells for patients to be treated.

Human NSCs can be obtained from brains and from human embryonic stem cells (hESCs) by a step-wise differentiation procedure [5-8], and such hESC-derived NSCs are usually cultured as NS-like aggregates. However, the NS-like aggregates, also called neuroectodermal spheres (NESs; [9]), differ from the NSs in that NESs have a distinctive radial cluster of columnar epithelial cells, called a rosette [8,10]. The rosette resembles a developing neural tube and contains multipotential neuroprogenitor cells that have a similar expression profile as the neuroepithelial cells in the neural tube. Analyses of microarray data revealed that brain-derived NSCs and hESC-derived NSCs were shown to express distinct groups of genes and, nonetheless, they did share many properties involving NSC markers [11,12]. In addition, the brain-derived NSs generally exhibit specific regional markers along with dorso-ventral and antero-posterior axes and, of them, hESC-derived NESs tend to preferentially express markers of anterior neural ectoderm [12]. Together, NESs might be assumed to mimic the pattern of *in vivo *neurogenesis to a degree [13].

It is well known that Notch signaling has a role in deciding cell fates during development [14]. With regard to neural development, Notch signaling also has an important role in the maintenance of neural stem-cell features. Notch1, Presenilins and RBP are key Notch signaling molecules – a receptor, a regulator and a co-effector, respectively. In the fetal brains of Notch1^-/-^, Presenilins^-/-^, or RBP-Jκ^-/- ^mice, NSC levels were shown to be completely depleted [15]. Also, inactivation of Notch-regulated genes such as *Hes1 *and *Hes5 *induced a premature neuronal differentiation during brain development [16]. Studies with Presenilin-deficient mice have shown that Notch signaling is necessary to maintain all NSCs, regardless of their locations in the brain or age of the mouse [17]. Several studies have provided important findings about the roles of Notch signaling in neural development; unfortunately, however, most of these studies have investigated the NSCs of mice or other laboratory animals rather than humans, mainly owing to the difficulties associated with obtaining human brain samples. Therefore, information on human NSCs is scarce, which prompted us to focus on neuroectodermal spheres (NESs) which are derived from human embryonic stem cell (hESC) and densely inhabited by NSCs. hESC-derived NSCs could possibly replace the rare human NSCs [4], which highlights the importance of the study to characterize the complicated, web-like molecular events, including Notch signaling, that occur in the *in vitro*-produced NESs.

In this study, we investigated the role of Notch signaling in hESC-derived NESs. We first verified that hESC-derived NESs had features similar to neurospheres derived in vivo. We demonstrated that Notch-related molecules were expressed at higher levels in the NESs than in the hESC-derived embryoid bodies. Furthermore, when Notch signaling was inhibited by a specific inhibitor for the γ-secretase, the rosette folds were not visible, and the self-renewing activity and the proliferative potential were significantly reduced in the resulting NESs. These observations indicate that Notch signaling is active in the NESs, and, to our knowledge, this, along with a recent paper by Elkabetz *et al. *[12], is the first description about the role of Notch signaling in maintaining self-renewal of NSCs derived from hESCs.

## Methods

### Human embryonic stem cell (hESC) culture

CHA-hES3 [18] was maintained on mitomicin C-treated STO feeder cells (ATCC, Manassas, Virginia, USA). H9 [19] (WiCell, USA) was maintained on γ-irradiated mouse embryonic fibroblasts in gelatin-coated culture dishes at 37°C, 5% CO_2 _in air. These hESCs were sub-cultured by mechanical section using a hand-made glass pipette. Human ESCs were cultured in DMEM/F12 containing 20% serum replacement, 0.1% non-essential amino acids, 0.1 mM β-mercaptoethanol, 100 U/ml penicillin-streptomycin and 4 ng/ml basic fibroblast growth factor (bFGF)(All from Invitrogen, Carlsbad, USA). Culture media were replenished everyday. Our research was performed under ethical approval from the Institutional Review Board (IRB) at KRIBB.

### Generation and culture of Neuroectodermal spheres (NESs) from hESCs

Human ESC colonies were dissected into 500 μm squares by tissue chipper [20] or ESCD with 500 μm pattern [21], transferred to plastic Petri dishes containing EB medium (hESC medium plus 10% serum replacement without bFGF), and cultured for 7 days. EB medium was then replaced to neuroectodermal sphere medium (NSM; DMEM:F12(1:1 in volume), B27 supplement (1:50), N2 supplement (1:100), 100 U/ml penicillin-streptomycin, 20 ng/ml bFGF, 20 ng/ml human epidermal growth factor (EGF) (all from Invitrogen) and 10 ng/ml human leukemia inhibitory factor (LIF, Sigma, St. Louis, MO)). A half medium was refreshed in every 48 hours. NESs were sub-cultured using McIlwain tissue chopper (Mickle Engineering, Gomshall, Surrey, UK) when they were grown to 500 μm in diameter [22]. Volume of NESs was measured by the formula for the volume of sphere, (4/3)πr^3^; radiuses of individual spheres were determined by taking mean length of long and short axis.

### RT-PCR analysis

Total RNA was isolated from hESCs, EB and NESs using RNesay kit (Qiagen, Valencia, UAS) and reverse-transcribed into cDNA with Superscript First Strand Synthesis System (Invitrogen) using oligo-d(T) primer as described in the manufacture's instructions. As a reference, the transcripts of *GAPDH *(for glyceraldehydes-3-phosphate dehydrogenase) or *β-actin *gene were amplified. Sequence information of primers and the lengths of amplified products are seen in Additional file 1. Primers for amplifying CNS marker genes are listed elsewhere [23]. Amplification conditions were as follows: single cycle of 94°C for 5 min followed by 30 – 35 cycles of 94°C for 30 s, 56–60°C for 30 s and 72°C for 30 s, and the final single cycle of 72°C extension for 7 min. Products were analyzed on 1.5% agarose gel and visualized by ethidium bromide staining.

### Immunocytochemistry

Floating NESs were plated on the matrigel (BD Biosciences, San Jose, CA)-coated dishes. The NESs attached were fixed with 10% formalin solution (containing 4% formaldehyde, Sigma) for 20 min followed by permeabilization for 30 min in PBS containing 0.1% Triton X-100. After blocking with 4% normal donkey serum (Molecular Probes, Eugene, OR, USA) for 1 hour, the samples were incubated with following primary antibodies at 4°C overnight: antibodies for Nestin (1:200, Chemicon, Temecula, CA), PAX6 (1:50, DSHB, Iowa City, Iowa, USA), NOTCH1 (bTAN20, 1:50, DSHB), DLL1 (1:200, Santa Cruz Biotechnology, CA, USA), TUJ1 (1:500, Covance, Madison, Wisconsin, UAS), JAG1 (1:50, Santa Cruz Biotechnology), N-Cadherin (1:50, Santa Cruz Biotechnology). Primary antibodies were detected by using Cy2- or Cy3-conjugated Donkey anti-Goat, anti-rabbit or anti-mouse secondary antibodies (Jackson laboratories, West Grove, PA, USA) for 45 min at room temperature. After reaction with secondary antibodies, the cells were stained with 100 nM DAPI (4',6-diamidino-2-phenylindole)(Molecular Probes) for 5 min, and mounted. Fluorescence-labeled NESs were viewed under an IX51 Olympus fluorescence microscope (Olympus, Japan) or Axiovert 200M equipped with ApoTom (Carl Zeiss, Gottingen, Germany).

### Neuroectodermal sphere re-forming Assay

NESs were dissociated with 2 mg/ml collagenase into single cells and cultured in NSM containing 0.1% DMSO or 5 μM DAPT for 17~18 days at a density of 1 × 10^5 ^cells/ml. Fifty percent of medium was replaced every 4~5 days. NESs with sizes more than 50 μm were counted.

### BrdU incorporation assay

Cells cultured in the NSM were treated with 10 μM 5-bromo-2'deoxyurine (BrdU; Roche Molecular Biochemicals, Sussex, UK) for 24 hours. Spheres were dissociated with collagenase and plated on the matrigel-coated coverslip for counting. Cells were fixed with formalin solution 10% (Sigma) for 15 min followed by permeabilization for 30 min in PBS containing 0.1% Triton X-100. DNA denaturation were performed by 2N HCl for 10 min and neutralized with 0.1 M Sodium tetra-borate for 10 min. Following procedures were the same as immunocytochemical method above mentioned. Genome-integrated BrdUs were detected using anti-BrdU antibody (1:200, BD pharmingen, San Diego, USA) and Cy3 conjugated anti-mouse secondary antibody. The proportion of BrdU positive cells relative to total cells counted was estimated under a fluorescent microscope.

### Trypan blue staining

NESs cultured in the NSM containing 0.1% DMSO or 5 μM DAPT for 4 days were dissociated with 2 mg/ml collagenase into single cells. An equal volume of Trypan blue stain solution (0.4%, Invitrogen) was added to the cell suspension. After 5 min, trypan blue stained cells and total cells were counted using a hemacytometer under the IX51 Olympus inverted microscope (Olympus).

### Quantification of TUJ1-positive cells in NESs

After 4 day culture in the NSM containing 0.1% DMSO or 5 μM DAPT, NESs were dissociated into single cells with 2 mg/ml collagenase and allowed to attach on the matrigel-coated coverslip. After immunostaining either with Nestin or TUJ1 antibody, the proportion of Nestin- or TUJ1-positive cells relative to the whole cells counted was calculated.

### Western blot analysis

Antibodies against Jagged1 (JAG1, Santa Cruz Biotechnology), Delta-like-1 (Dll1; Santa Cruz Biotechnology), cleaved Notch1 (NICD; Cell Signaling Technology, Beverley, MA USA), Nestin (Chemicon), TUJ1 (Covance, Richmond, CA), MAP2 (Chemicon), S100 (Abcam, Cambridge, UK), GFAP (Novus Biologicals, Littleton, CO, USA), NG2 (Chemicon), CNPase (Chemicon), HES1 (Chemicon) and HES5 (Sigma) were used for Western-blot analyses. For protein extraction, cells were lysed in a buffer containing 20 mM HEPES, 50 mM NaCl, 10% glycerol, 0.5% Triton X-100 and 2% β-mercaptoethanol. Concentrations were determined by the Bradford method. The protein samples (20 μg) were separated by 6%, 8% and 15% SDS-PAGE and transferred to a nitrocellulose membrane (Schleicher and Schuell Inc., Keene, NH, USA) with Tris-glycine-methanol buffer (25 mM Tris, 192 mM glycine and 20% methanol). After blocking with the TBS buffer (10 mM Tris-HCl pH 7.5, 150 mM NaCl) containing 5% non-fat dry milk and 0.1% Tween20, the membrane was incubated with primary antibodies, followed by incubation with horseradish peroxidase-conjugated goat antibody to rabbit IgG (Santa Cruz Biotechnology), and developed with enhanced chemiluminescence reagent (Amersham, Piscataway, NJ, USA).

## Results and Discussion

### Derivation and characterization of neuroectodermal spheres from human embryonic stem cells

**Figure 1:**
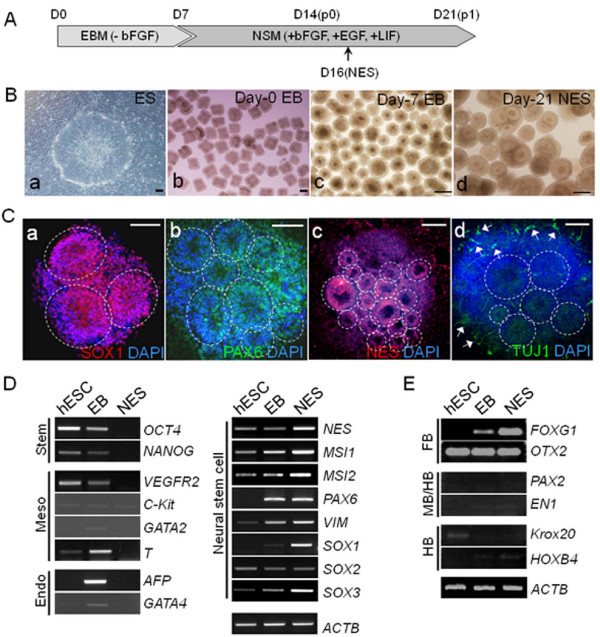
**Controlled derivation of neuroectodermal spheres from human embryonic stem cells**. (**A**) Schematic showing neuroectodermal sphere (NES). By a simple medium change without an attachment step, embryoid bodies (EBs) could be differentiated to NESs harboring neuroprogenitor cells. EBs were grown in EBM for a week and then transferred to NSM supplemented with growth factors. The first subculture was performed one week later (D14) and, about two days later (D16), rosette-containing NESs appeared. The NES samples we used were D21 NESs, if not otherwise indicated. (**B**) Photographs of differentiating cell clumps at indicated times. Human embryonic stem cell (hESC) colonies (**a**) were divided into regular-sized (500 μm in length) clumps (**b**) using a chopper. Floating EBs at day 7 (**c**) are shown. NESs at day 21 have prominent rosette-like folded structures in the spheres (**d**). We piled up EBs and NESs in single spots before taking pictures. (**C**) Expression of neural stem cell (NSC) markers in NESs. NESs were allowed to attach to culture equipment and were stained either for SOX1 (**a**), PAX6 (**b**), Nestin (NES, **c**) and TUJ1 (**d**). TUJ1-positive neurites are scattered, usually around the boundaries of NES clumps (arrows). Boundaries of rosettes are indicated by dotted circles. (**D**) RT-PCR for various marker genes of different cell lineages. NSC marker genes are abundantly transcribed in hESC-derived NESs (**right panel**). Other lineage markers such as those of ESCs (Stem), mesoderm lineage cells (Meso) and endoderm lineage cells (Endo) are not preferentially expressed in NESs (**left panel**). β-*Actin *(*ACTB*), internal control. (**E**) RT-PCR analysis for markers of anterior regional identity (FB; FOXG1 and OTX2), mid-hind brain markers (MB/HB; PAX2 and EN1), and posterior CNS markers (HB; KROX20 and HOXB4). Scale bars, 200 μm in **B **and 100 μm in **C**; EBM, embryoid body medium; NSM, neurosphere medium; bFGF, basic fibroblast growth factor; EGF, epidermal growth factor; LIF, leukemia inhibitory factor.

We derived NESs containing neuroprogenitors from the hESCs CHA3-hESCs [18] and H9 [19]. Figure 1A shows the procedure and timetable of NES preparation. We used a tissue chopper [20] or embryonic stem cell divider (ESCD) [21] to prepare embryoid bodies (EBs); both of these approaches produce regular-sized, square clumps of hESCs (500 μm in length, Figure 1Bb). These clumps were cultured in EB medium (EBM) for 7 days (Figure 1Bc) and transferred to NES medium (NSM) to further differentiate into NESs. Neural rosettes, which are structures with neural tube-like folds and central cavities surrounded by rings of small columnar cells [8], appeared about 2 days after the first subculture (Figure 1A, D16(NES)). This was characteristic of NESs (Fig 1Bd).

The hESC-derived NESs attached to the Matrigel-coated culture dish were immunostained for neural stem cell (NSC) markers such as SOX1, PAX6 and Nestin. The rosettes of various sizes were positively stained for all these NSC markers (Figure 1C). In addition, the hESC-derived NESs were stained for a neuronal marker TUJ1, we found TUJ1-positive neurites sporadically scattered around the boundaries of NES clumps. Flow cytometry showed that more than 95% in both CHA-hES3- and H9-derived NESs were positively stained for the neural precursor cell surface marker PSA-NCAM [24]**(data not shown**). When analyzed at the transcriptional level, the NESs showed increased expression levels of NSC marker genes such as *NES, MSI1 *and *2, PAX6, VIM, SOX1*, and *SOX3*, whereas none of the mesoderm-lineage markers (Meso; *VEGFR2, C-KIT, GATA2 *and *T*) or the endoderm-lineage markers (Endo; *AFP *and *GATA4*) were transcribed in a NES-specific manner (Figure 1D). The transcripts for the ESC marker genes (Stem) *OCT4 *and *NANOG*, were undetectable in the NESs. The expression patterns of these NSC markers are similar to recent reports; for example, *PAX6 *expression continued in 7-day-old EBs, whereas *SOX1 *expression began only after NES formation [10]. RT-PCR results showed that anterior CNS markers such as FoxG1 and Otx2 were more expressed in the NESs than mid-hindbrain markers such as Pax2 and En1 and markers of posterior CNS fate such as Krox20 and HoxB4 [23] (Figure 1E). This result agreed with a recent report [12], suggesting that in the absence of extrinsic patterning cue, NESs acquire markers defining anterior CNS identity. Taken together, these morphological, immunocytochemical, and molecular-level results demonstrate that the hESC-derived NESs are suitable as an *in vitro *model of human *in vivo*-derived neuroprogenitors.

### Components of Notch signaling were up-regulated in hESCs-derived NESs

**Figure 2:**
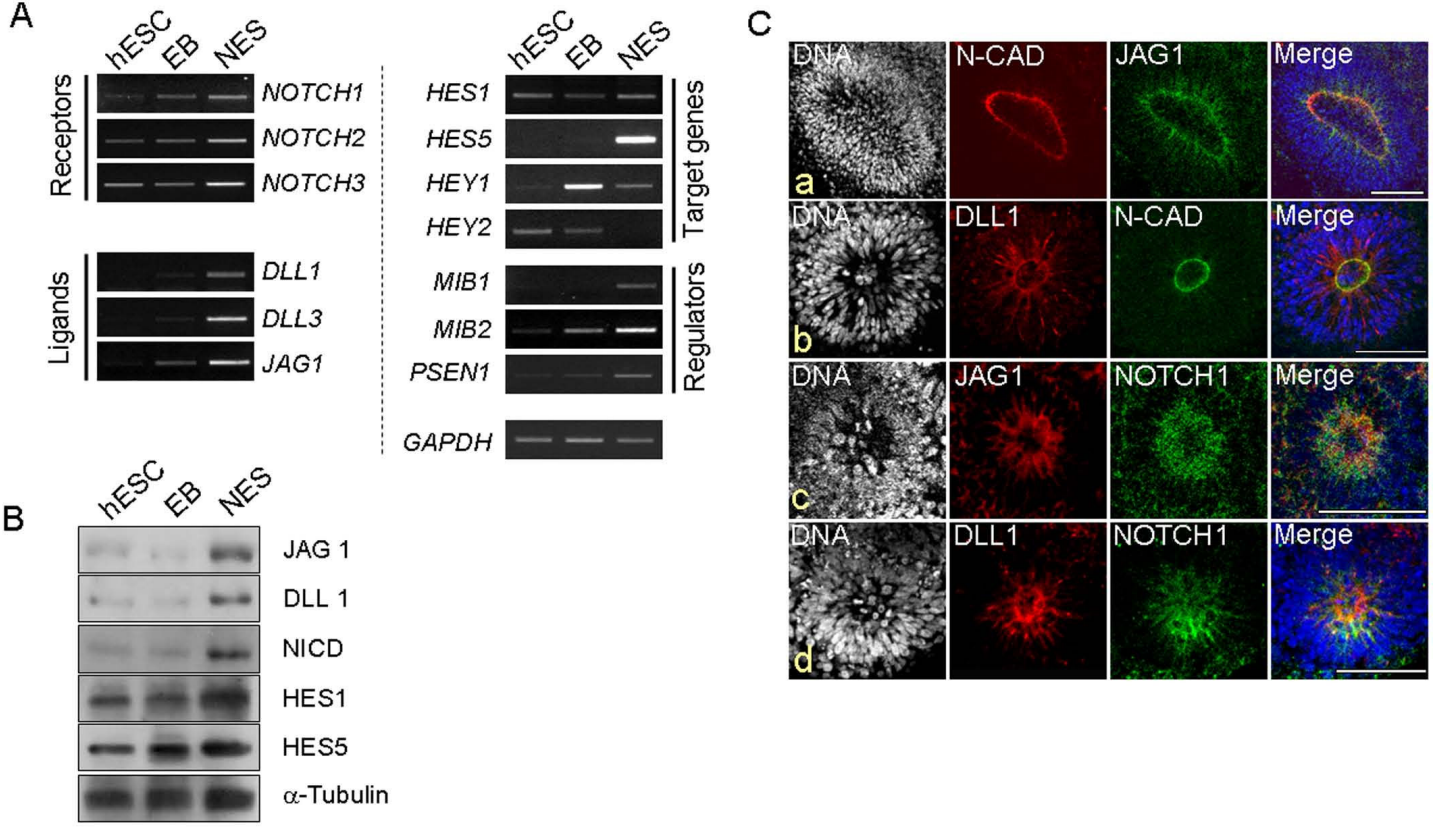
**Notch signaling is active in neuroectodermal spheres derived from human embryonic stem cells**. (**A**) Transcriptional expression of Notch signaling members in neuroectodermal spheres (NESs). All components functionally related with Notch signaling pathway were up-regulated in the NES samples. *NOTCH4 *was not expressed in all samples (data not shown). *HEY1 *and *HEY2 *were not preferentially expressed in the NESs, because they are regulated by Notch signaling but are associated with vascular development [30]. *GAPDH*, loading control. (**B**) Western-blot analyses of Notch signaling molecules in the NESs. All proteins of the Notch signaling pathway examined were expressed in hESCs, EBs and NESs but all were most abundant in the NES protein extracts. NICD, NOTCH Intracellular Domain (or cleaved Notch1). α-TUBULIN, loading control. (**C**) Immunostaining of the rosettes for the Notch ligands (JAG1 and DLL1) and receptor (NOTCH1). Rosettes structures derived from hESC-derived NESs were visualized using the rosette marker N-cadherin (N-CAD) and shown to locate to the inner rims of the rosettes. Both JAG1 (**a **and **c**) and DLL1 (**b **and **d**) transmembrane ligands are localized in the regions where N-cadherin signals exist. Anti-NOTCH1 antibody, which recognizes both NICD and C-terminal cytoplasmic domain of membrane-bound NOTCH1 receptor simultaneously, generates a rather diffused signal (**c **and **d**); nevertheless, its location at the luminal side of rosette is evident. Scale bars, 100 μm.

Notch signaling has been proposed to maintain the property of neuroprogenitors obtained from brain samples [17,25,26]. To investigate the role of Notch signaling in the NESs, we first profiled expressions of Notch signaling genes. RT-PCR was used to show that transcripts for receptors *NOTCH1, NOTCH2 *and *NOTCH3 *were present at slightly increased levels in the NESs compared with hESCs and EB (Figure 2A). The NOTCH ligands *DLL1, DLL3 *[15] and *JAG1 *[27] were abundantly expressed in the NESs. However, the *NOTCH4 *transcript was not detected either from hESCs or NESs (data not shown); this is in agreement with a previous report [28]. Both *HES1 *and *HES5*, which are regulated by Notch signaling and involved in neurogenesis [29], were markedly expressed in the NESs; they were also expressed in undifferentiated hESCs in a small amount as observed before (13, 25, 26, 42, 43). *HEY1 *and *HEY2*, which are also regulated by Notch signaling but are associated with vascular development [30], were not preferentially expressed in the NESs. *MIB1 *and *MIB2 *[31], which are required for ligand activation, and *PSEN1*, which is the catalytic subunit of γ-secretase that cleaves NOTCH receptor to release the major signal transmitter, NOTCH intracellular domain (NICD) [32], were also shown to be expressed at high levels in the NESs. Expression of Notch signaling molecules were also confirmed at the protein level. In agreement with the RT-PCR results, Notch signaling pathway proteins such as JAG1 and DLL1 and NICD and the target gene products HES1 and HES5 were abundant in hESC-derived NESs (Figure 2B). When the attached NESs were stained for JAG1 or DLL1, each of which is a transmembrane NOTCH ligand, JAG1 and DLL1 signals (Figure 2Ca and 2Cb, respectively) were shown to be enriched in the rosettes and were at particularly high levels in the inner circle of the rosette structures, facing the luminal side (Figure 2C). We also found that NOTCH1 receptor was also localized at the luminal side of rosettes together with JAG1 (Figure 2Cc) and DLL1 (Figure 2Cd). It has been shown that JAG1 specifically expressed neuroepithelial cells in apical termini of fetal brain [33]. The JAG1 staining pattern was in agreement with N-cadherin signal and formed a belt- or adherens junction-like signal. In a similar vein, it was reported that in the ventricular zone, DLL1 is linked with adherens junction through interaction with MAGI1 at the apical termini of processes to activate Notch on neighboring cells in the developing central nervous system [34]. Owing to the stacking property of the cells facing the luminal side of rosettes, it is difficult to find out whether both notch ligand and receptor are expressed within the same cells. Nevertheless, it is worth to notice a recent study that both Notch1 and Dll1 co-existed in ependymal cells [35]. Together, these findings indicate that Notch signaling has a greater role in cellular function in the hESC-derived NESs than in the hESCs and EBs [36]. On the whole, the gene expression profile of the hESC-derived NESs corresponded with the previously reported results using *in vivo *NSCs [15,16,26,37,38], which indicates that the neuroprogenitors in the NESs/rosettes express many of the same genes that are expressed in neuroepithelial cells of the neural tube.

### Inhibition of Notch signaling leads to a loss of the stem cell characteristics from the neuroectodermal spheres

**Figure 3:**
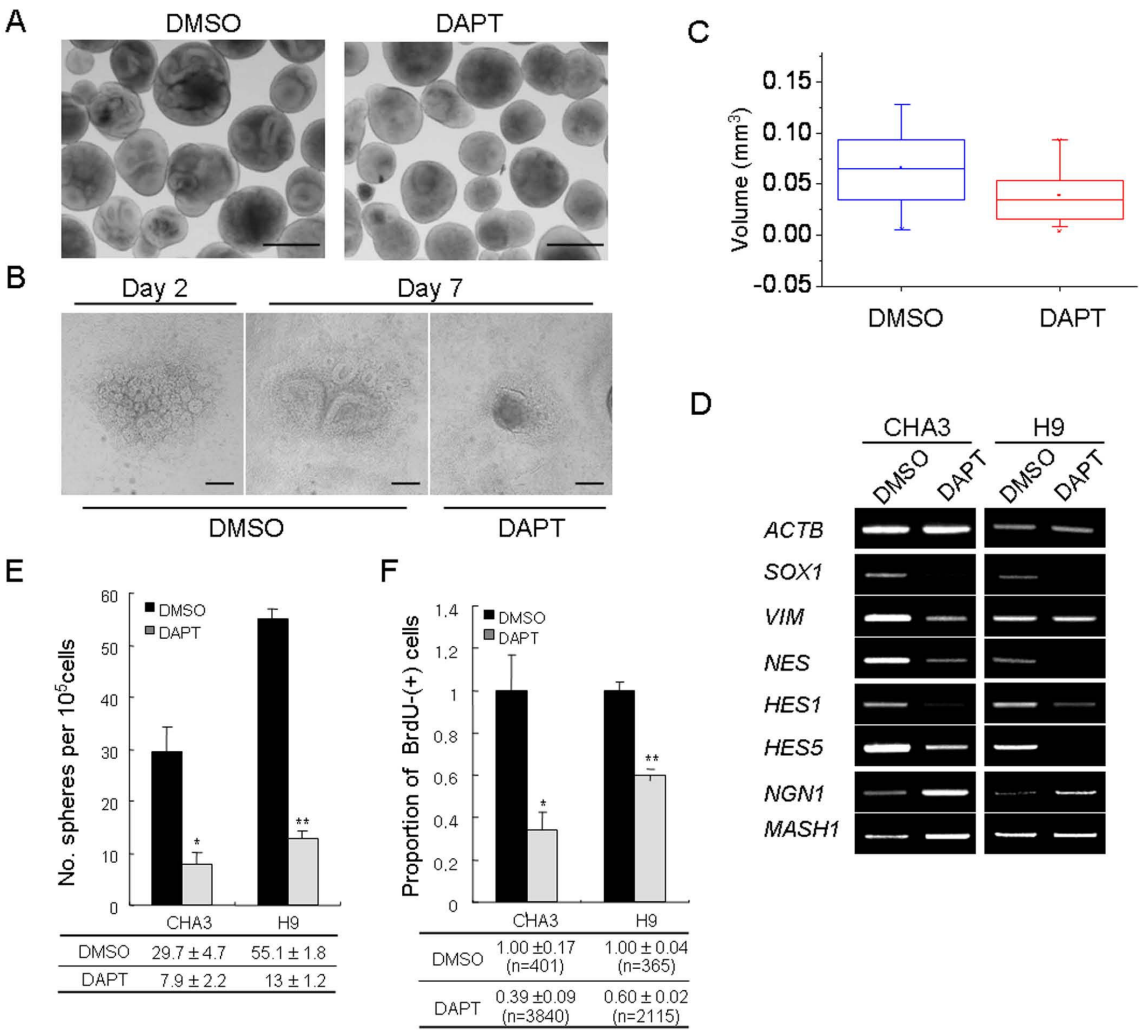
**Notch inhibition disturbs the maintenance of stem cell characteristics of neuroprogenitor cells in the hESC-derived neuroectodermal spheres**. (**A**) Disappearance of the rosette structure with DAPT treatment. Neuroectodermal spheres (NESs) derived from CHA-hES3 were cultured with or without DAPT (5 μM) for 4 days. Rosette-like folded structures in NESs were markedly reduced with DAPT treatment. CHA3 hESCs were used. Scale bars, 500 μm. (**B**) DAPT-induced loss of rosette structures. DAPT treatment removes rosettes from attached NES clumps whereas the DMSO control group still contains rosettes. H9 hESCs were used. Scale bars, 200 μm. (**C**) Reduction in volume of the DAPT-treated NESs. The volume of NESs after DAPT treatment for 4 days was measured. Control group (black) was estimated as 0.066 ± 0.042 mm^3 ^(mean ± standard deviation (SD); n = 44) and the DAPT group (red) was 0.039 ± 0.027 mm^3 ^(n = 58). The difference between the two groups was significant (*p *< 0.001). In the box plot, box percentage and mean values (open squares) are indicated. Whiskers range from 5–95%. 'x' in each box indicates an upper and lower extreme value. (**D**) RT-PCR results. Neural stem cell marker genes (*SOX1*, *VIM*, and *NES*) and Notch-regulated target genes (*HES1 *and *HES5*) showed reduced expression levels in DAPT-treated NESs derived from CHA3 and H9 hESCs. However, expression of the pro-neuronal genes, *NGN1 *and *MASH1*, were reversely increased by DAPT-treatment. (**E**) Neuroectodermal sphere-reforming assay. NESs were dissociated into single cells and cultured for 17–18 days with (gray) or without (black) DAPT (5 μM). Among the newly formed NESs, those larger than 50 μm diameter were counted. The values denote mean ± standard error and there are significant differences between the control and the DAPT-treated groups (*p *< 0.05). Experiments were independently performed three times. (**F**) BrdU incorporation assay. The proportion of mitotically active cells was reduced in DAPT-treated NESs. The statistical values denote mean ± standard error, and 'n' indicates the number of cells counted. Data were obtained from more than three (CHA3, *p *< 0.05) or five (H9, *p *< 0.005) independent experiments.

After demonstrating that Notch signaling is active in the NESs, we investigated the potential role of Notch signaling in the NESs derived from hESCs. We treated the NESs with the Notch signaling inhibitor, *N*-[*N*-(3,5-difluorophenacetyl)-L-alanyl]-*S*-phenylglycine-*t*-butyl ester (DAPT), which is known to specifically bind to Presenilin-1 (PS1) and inhibit γ-secretase activity [28]. Surprisingly, in both CHA3 and H9 cell lines, the treatment with 5 μM DAPT removed the rosette structures from most of the floating (Figure 3A, CHA3 data only shown) and attached NESs (Figure 3B, H9 data only shown). The volume of floating NESs after DAPT treatment was 0.039 ± 0.027 mm^3^; (mean ± standard deviation (SD); n = 58), and this value was only 59% of the value of DMSO control (0.066 ± 0.042 mm^3^; n = 44; *p *< 0.001) (Figure 3C). DAPT treatment did not cause damages such as cell death, as evidenced by the observation that trypan-blue staining showed that the survival rates were almost equal between DMSO only (97.7 ± 0.4, mean ± SD; n = 659) and DAPT (98.9 ± 1.3; n = 706) groups. Considering that the rosette contains neuroprogenitor cells reside [8], the reduced numbers of rosette structure in the NESs after treatment with a Notch inhibitor could indicate a reduction in the neuroprogenitor population. In agreement with this, RT-PCR results from the DAPT-treated NESs derived from both hESC cell lines showed a marked reduction in the expression levels of various NSC marker genes and Notch-regulated target genes such as *HES1 *and *HES5 *(Figure 3D). *NGN1*, which is suppressed by *HES5 *expression, were de-repressed in the DAPT-treated NESs in both CHA3 and H9 hESCs. *MASH1 *is another target gene that is negatively regulated by HES5, but its de-repression after DAPT treatment was not as immediate as that of NGN1.

To assess the proliferating capacity of neuroprogenitors in the Notch signaling-inhibited NESs, we performed NES-reforming assay [15,26,39]. NESs were enzymatically dissociated into single cells and allowed to re-construct sphere colonies in NSM with or without DAPT. As shown in Figure 3E, the number of emerging spheres in the DAPT-treated cells was reduced to 25% that of the control group in CHA3 (of 10^5 ^cells plated, 29.7 ± 4.7 (mean ± standard error) and 7.9 ± 2.2 cells in DMSO control and DAPT-treated cells, respectively; *p *< 0.05) and H9 (55.1 ± 1.8 cells for control *vs *13.0 ± 1.2 cells for DAPT-treated group; *p *< 0.005) cell line s. The reformation frequency of hESC-derived sphere colony has not previously been estimated with hESC-derived NESs, making it impossible to compare the reformation efficiency, but it seems to be about ten-fold less than the frequency (2.4%) of mouse neuroprogenitors derived *in vivo *[40]. In addition, results of the bromo-2'-deoxyuridine (BrdU) incorporation assay showed that DAPT treatment reduced the proportion of replicating cells to 39% of the control group for CHA3 hESCs or 60% of the control group for H9 hESCs (Figure 3F). Together, these results show that the Notch signaling is mainly involved in the maintenance of rosette structures, the biochemical roles of which are probably linked to maintenance and self-renewal of the neuroprogenitor population in NESs.

### Inhibition of Notch signaling drives neuroectodermal spheres to differentiate into neuronal cells

**Figure 4:**
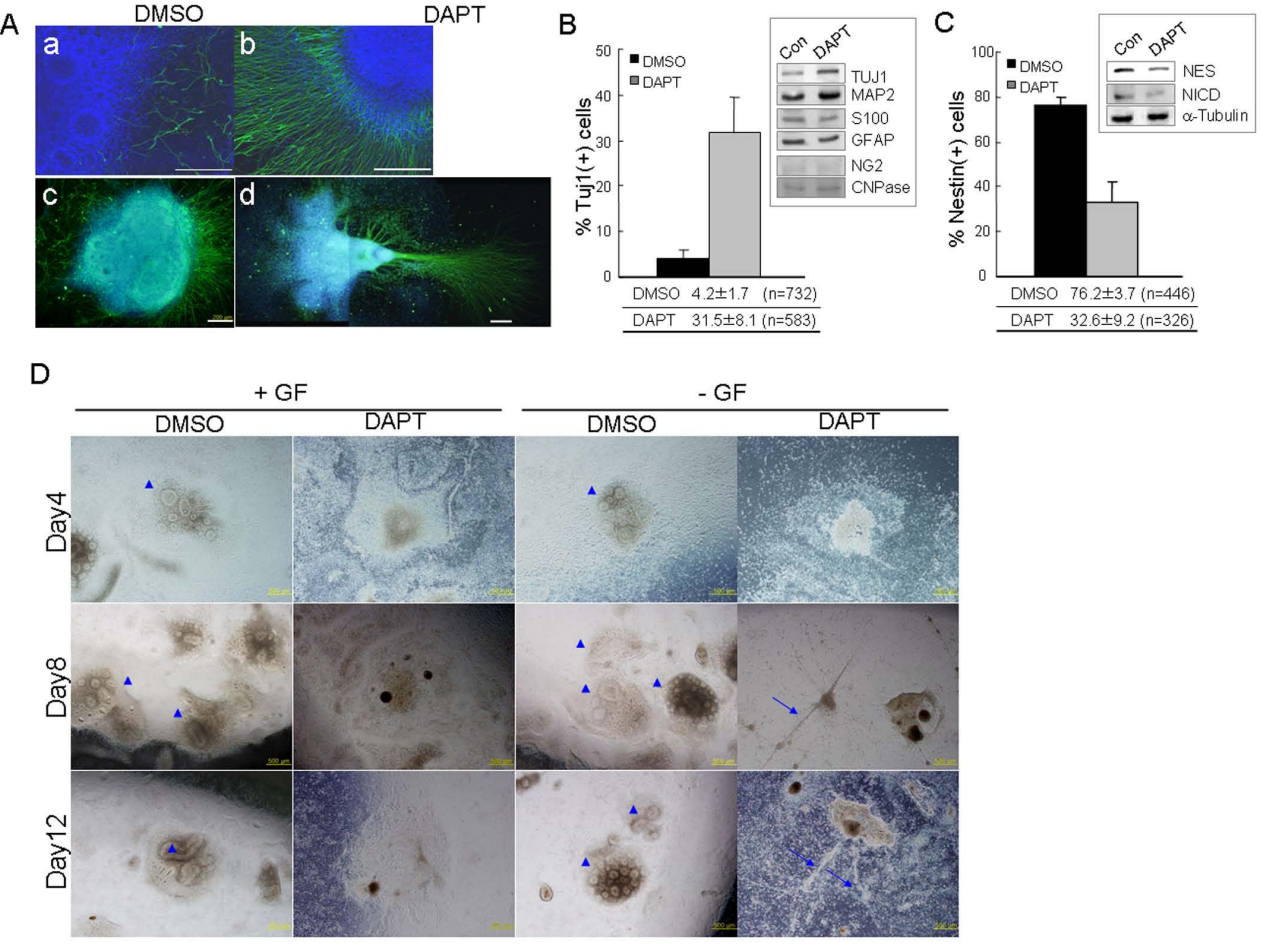
**Inhibition of Notch signaling directs cells of neuroectodermal spheres to differentiate into neuronal cells**. (**A**) Immunostaining of attached NESs for TUJ1. NESs derived from H9 hESCs were treated with DMSO or DAPT for 4 days and stained for TUJ1 (green). Images of NESs are shown in parts in **a **and **b **or as whole clumps in **c **and **d**. In **d**, two consecutive pictures separately taken are combined. (**B-C**) Measurement of TUJ1-positive cells after DAPT treatment. NESs cultured in the NSM containing DMSO (solid bars) or DAPT (gray bars) for 4 days were dissociated into single cells and attached on the matrigel-coated coverslip before immunostaining for TUJ1 (**B**) and Nestin (**C**). The number of positively stained cells relative to DAPI-positive cells was calculated. The values denote mean ± standard deviation and there are significant differences between the control and the DAPT-treated groups (*p *< 0.005 for TUJ1 and *p *< 0.001 for Nestin). Experiments were independently performed five times and 2087 cells in total were counted. Results of Western-blot analyses of DMSO control and DAPT treated samples are shown in the boxes, in agreement with the results of cell counting. TUJ1 and MAP2 are neuronal markers, and S100, GFAP, NG2 and CNPase are glial markers. α-Tubulin, a control for the bands in B and C. (**D**) Enrichment of neurite bundles by withdrawal of growth factors. The rosettes structures are clear, regardless of GF presence (arrowheads) but not visible in the DAPT-treated samples. Neurite formation was markedly accelerated in the DAPT-treated NESs after withdrawal of growth factors such as bFGF, LIF and EGF (arrows). Such neurite bundles are rarely seen in DAPT-treated NESs in the presence of growth factors. Scale bars, 200 μm in **A **and 500 μm in **D**.

We also investigated the effects of Notch inhibition by DAPT. RT-PCR analyses for the expressions of neuroprogenitor marker genes and Notch-related genes showed that DAPT treatment of NESs resulted in a marked change in the gene expression profile (Figure 3D). In general, Notch inhibition induces neuroprogenitor cells to differentiate to neuronal cells in vertebrate and invertebrate (for review, see [3]). We examined whether the hESC-derived NESs displayed a similar trend of differentiation. Immunostaining of the DAPT-treated NESs for 4 days showed that neurite formation was markedly increased compared with the DMSO control, as shown by α-TUJ1 antibody staining (Figure 4A). We counted the number of TUJ1-positive cells after dissociation of NESs into single cells. The proportion of TUJ1-positive cells was 4.2 ± 1.8% (mean ± standard deviation) and 31.5 ± 8.1% in DMSO control and DAPT-treated NESs, respectively (*p *< 0.005; Figure 4B). As a reference, the proportion of Nestin-positive cells was 76.2 ± 3.7%and 32.6 ± 9.2% in DMSO control and DAPT group, respectively (*p *< 0.001; Figure 4C). Western-blot analyses showed that expression levels of neuronal markers such as TUJ1 and MAP2 were increased in DAPT-treated NESs, while not the levels of glial markers such as GFAP, S100, NG2 and CNPase (inset in Figure 4B). These results indicate that DAPT-mediated Notch inhibition enriches neuronal cell population in the NESs.

Moreover, withdrawal of growth factors (GFs) such as fibroblast growth factor 2 (FGF2), leukemia inhibitory factor (LIF) and epidermal growth factor (EGF) [41] from the NSM furthered differentiation of the hESC-derived NESs into neuronal cells (Figure 4D), agreeing with previous reports [42,43]. In the absence of GFs, DAPT treatment resulted in accumulation of neurite bundles about NESs (see arrows in Day8 and Day12), in contrast to the samples treated with both GF and DAPT in which neurite bundles were hardly detected. These results coincided with the RT-PCR result in Figure 3D where Notch inhibition resulted in silencing *HES1 *and *HES5 *genes, which in turn continued to de-repress pro-neuronal genes such as *NGN1 *and *MASH1 *[29,38]. Meanwhile, the GF-negative DMSO control at day 8 and 12 still possessed rosettes, the sizes of which, however, were tiny compared with those of GF-positive DMSO control. This leads to a speculation that small-sized rosettes are supposed to be gradually fused together to large-sized ones and, in this process, GFs play roles in facilitating the presumable fusion event among individual rosettes. Together, our results indicate that inhibition of Notch signaling disturbs the self-renewal of neuroprogenitors in the hESC-derived NESs and leads ultimately to differentiation to neuronal cells. However, we should keep in mind that the γ-secretase that is inactivated by DAPT not only cleaves Notch receptors but also other proteins (for review, see [44]). For confirmation that Notch inhibition indeed directs neuronal differentiation, evidence needs to be provided supporting that the DAPT effects on rosette structure can be rescued with exogenous NICD expression in DAPT-treated cultures, or be mimicked by knockdown experiments for NICD or RBP.

NSCs have considerable therapeutic values in cell-replacing regenerative treatment of currently incurable neural diseases. In addition, unlimited supply of functional human neurons is only possibly from NSCs, and this would allow a fast and effective high throughput screening for neural disease therapies. Human ESCs are undoubtedly the preferred source of NSCs. We have developed a simple method for deriving NSCs or neuroprogenitors from hESCs, with an emphasis on minimizing the degree of variation among individual EBs and sphere colonies through size regulation (Figure 1A). The use of sub-culture devices such as tissue chopper [20] or ESCD [21] allowed us to obtain EBs with regular sizes (Figure 1B) that form homogeneous NESs. Our method of NES derivation has the advantages of a short culture period, thus avoiding additional attachment and selection steps [10]. This markedly simplifies the current NES derivation procedures [6,8,45,46] without reducing the efficiency, which is necessary for the practical application of hESC-derived NSCs to cell therapeutics and drug screening. Our NES derivation protocol is similar to a recently reported protocol [5].

In vertebrate, activation of Notch signals inhibits neuronal differentiation and maintains the stem-cell characteristics of NSCs or neuroprogenitors derived in vivo [3]. We investigated whether Notch signaling is active and therefore has a genuine role in the hESC-derived NESs, and we obtained several results. First, results of RT-PCR and Western-blot analyses showed that most of the known key components of the Notch signaling pathway such as receptors (NOTCH1, NOTCH2 and NOTCH3), ligands (DLL1, DLL3 and JAG1), and regulators (NICD, MIB1, MIB2 and PSEN1) were abundantly expressed in the NESs at the protein and mRNA levels (Figure 2A and 2B). Second, the expression levels of Notch signal members and the resulting target genes (HES1 and HES5) were increased in the NESs compared with those in the EBs. This was particularly true for the NICD, DLL1, JAG1 and HES1 (Figure 2B). Third, immunostaining of the NESs for the plasma-membrane-bound ligand JAG1 and DLL1 demonstrated that both are localized mainly to the cells comprising inner rims of the rosettes, rather than being expressed throughout the NESs (Figure 2C). Fourth, the treatment with Notch inhibitor DAPT removed rosette structures from both floating and attached NESs, and was associated with the reduction of NSC marker expressions and proliferation potential in the NESs (Figure 3). Finally, DAPT treatment induced neurite formation and increased expression of TUJ1 (Figure 4), indicating that Notch inhibition drives the NESs to differentiate preferentially into neuronal cells, in agreement with the observation that Notch-inhibited neuroprogenitor cells favor differentiation toward neuronal cells in vertebrate and invertebrate (for review, see [3]). Therefore, we concluded that Notch signaling actively functions in the NESs or, more specifically, in the rosettes, and that Notch signaling is responsible for maintenance of the stem-cell features of NSCs or neuroprogenitors in the rosettes. Therefore, our results indicate that the hESC-derived NESs or the neural rosettes are a good *in-vitro *model for neurogenesis *in vivo*.

## Conclusion

NSCs have considerable therapeutic values in cell-replacing-regenerative treatment of currently incurable neural diseases. hESCs are one of the best sources of NSCs or neuroprogenitor cells owing to their unlimited proliferation. In this study, we derived NESs containing neuroprogenitors from hESCs, and verified that these hESC-derived NESs were typical of neurospheres burying neuroprogenitors and were characteristic of activated Notch signaling. DAPT-induced inhibition of Notch signaling led to loss of the stem-cell characteristics from the NESs and drove them to differentiate into neuronal cells. These results are the first to demonstrate the roles of Notch signaling in hESC-derived NESs with biochemical features similar to those in neurospheres derived from animal brains, or fetal or adult human brains. Therefore, the hESC-derived NESs or neural rosettes are considered to be a good *in vitro *model for studying the neurogenesis that occurs *in vivo*. We believe that our results might aid further study of the mechanisms by which rosettes form and expand *in vitro*, how neuroprogenitor cells maintain their stem-cell-like characteristics in the cell culture environment, and the stem-cell characteristics that lead to asymmetric division.

## Authors' contributions

SMW designed the study, participated in data analysis and performed cell culture, RT-PCR and immunostaining. JK designed the study, participated in data analysis, performed immunostaining and wrote the manuscript. HWH contributed to the derivation of NES. JIC performed Western-blot analyses. MYS contributed to cell culture. SC contributed to RT-PCR. HMC contributed to the ES cell culture. YMH contributed to the design and manuscript draft. YKK designed the study, participated in Data analysis and wrote the manuscript. All authors read and approved the final manuscript.

## Supplementary Material

Additional file 1 **Supplemental table**. Primers of NOTCH signaling pathway related Human genes for RT-PCR. Click here for file
