# Supplementary material for: Notch signaling is required for maintaining stem-cell features of neuroprogenitor cells derived from human embryonic stem cells
Source: BMC Neurosci. 2009 Aug 17;10:97. doi: 10.1186/1471-2202-10-97 (PMC3224699; doi:10.1186/1471-2202-10-97)
Supplement: Additional file 1 — Supplemental table. Primers of NOTCH signaling pathway related Human genes for RT-PCR. [file 1471-2202-10-97-S1.doc]

**Additional file 1. Primers of NOTCH signaling pathway related Human genes for RT-PCR**

| *Genes* | Type | Sequence (5 → 3’) | size (bp) |
| --- | --- | --- | --- |
| *GAPDH* | Forward  Reverse | GAAGGTGAAGGTCGGAGTC  GAAGATGGTGATGGGATTTC | 226 |
| *β-Actin* | Forward  Reverse | CGCACCACTGGCATTGTCAT  TTCTCCTTGATGTCACGCAC | 200 |
| *NOTCH1* | Forward  Reverse | GCGGCCGCCTTTGTGCTTCTGTTC  GCCGGCGCGTCCTCCTCTTCC | 502 |
| *NOTCH2* | Forward  Reverse | TCGTGCAAGAGCCAGTTACCC  AATGTCATGGCCGCTTCAGAG | 532 |
| *NOTCH3* | Forward  Reverse | AAGTTACCCCCAAGAGGCAAGTGTT  AAGGAAATGAGAGGCCAGAAGGAGA | 335 |
| *NOTCH4* | Forward  Reverse | AGCAGACAAACTGCAGTGGA  CTGTTGTCCTGGGCATCTTT | 233 |
| *DLL1* | Forward  Reverse | CTGATGACCTCGCAACAGAA  CAGATCGGCTCTGTGCAGTA | 283 |
| *DLL3* | Forward  Reverse | GACCCTCAGCGCTACCTTTT  TACATCTTCAGGGCGATTCC | 249 |
| *DLL4* | Forward  Reverse | GCCTGGACAAGTCCAACTGT  CGCTGATATCCGACACTCTG | 176 |
| *JAG1* | Forward  Reverse | CCATTTCTGCTGAAGATATACGG  CACAGTTAAGACAGAGCTCAGCA | 194 |
| *HES1* | Forward  Reverse | CCAAAGACAGCATCTGAGCA  CATTGATCTGGGTCATGCAG | 373 |
| *HES5* | Forward  Reverse | CTCAGCCCCAAAGAGAAAAA  TAGTCCTGGTGCAGGCTCTT | 233 |
| *HEY1* | Forward  Reverse | ATGATTCCGTTTTTGCCTCA  CTCGCACACCATGATCACTT | 361 |
| *HEY2* | Forward  Reverse | ACCTCTCTCCACCTCTCTCTTGT  GGTTTATTGTTTGTTCCACTGCT | 225 |
| *NGN1* | Forward  Reverse | AGCTCACCAAAATCGAGACG  GGGCTACTGGGGTCAGAGAG | 226 |
| *NGN2* | Forward Reverse | CCGAGACCTTGGAGTTGAAG  CGTTTGCAATCGTGTACCAG | 250 |
| *MASH1* | Forward  Reverse | AAAGCTCTGCCAAGATGGAG  CTTGGGCGCTGACTTGTG | 248 |
| *MIB1* | Forward  Reverse | CTGATCTGAAATGTGTCCAGGAT  CTCAAACATTCCATCAGTCCATC | 194 |
| *MIB2* | Forward  Reverse | CTGGGTGCAGTGTCAACG  GTGGTTGGTGTAGCTCACGTC | 236 |
| *PS1* | Forward  Reverse | GGTGGAGCAAGATGAGGAAG  TGGCAGCATTCAGAATTGAG | 227 |
| *Oct4* | Forward  Reverse | GAAGGATGTGGTCCGAGTGT  GTGACAGAGACAGGGGGAAA | 243 |
| *Nanog* | Forward  Reverse | ACCAGAACTGTGTTCTCTTCCACC  GGTTGCTCCAGGTTGAATTGTTCC | 334 |
| *VEGFR2* | Forward  Reverse | AGCGATGGCCTCTTCTGTAA  ACACGACTCCATGTTGGTCA | 172 |
| *c-kit* | Forward  Reverse | TGACTTACGACAGGCTCGTG  AAGGAGTGAACAGGGTGTGG | 327 |
| *Brachyury* | Forward  Reverse | ACCCAGTTCATAGCGGTGAC  ATGAGGATTTGCAGGTGGAC | 216 |
| *α-FP* | Forward  Reverse | CCGAACTTTCCAAGCCATAA  TGGCATTCAAGAGGGTTTTC | 486 |
| *GATA4* | Forward  Reverse | TCCAAACCAGAAAACGGAAG  AAGACCAGGCTGTTCCAAGA | 352 |
| *Vimentin* | Forward  Reverse | GAGAACTTTGCCGTTGAAGC  TCCAGCAGCTTCCTGTAGGT | 170 |
| *Tuj1* | Forward  Reverse | ACCTCAACCACCTGGTATCG  GGGATCCACTCCACGAAGTA | 449 |
| *Nestin* | Forward  Reverse | AACAGCGACGGAGGTCTCTA  TTCTCTTGTCCCGCAGACTT | 220 |
| *Musashi1* | Forward  Reverse | TTCGGGTTTGTCACGTTTGAG  GGCCTGTATAACTCCGGCTG | 250 |
| *Musashi2* | Forward  Reverse | TTTGTAGGCGGGTTATCTGC  GCCATAGCTTGGAGCAAATC | 378 |
| *Pax6* | Forward  Reverse | ATGAGGCTCAAATGCGACTT  CATTTGGCCCTTCGATTAGA | 202 |
| *Sox1* | Forward  Reverse | GGGAAAACGGGCAAAATAAT  CCATCTGGGCTTCAAGTGTT | 376 |
| *Sox2* | Forward  Reverse | CATCACCCACAGCAAATGAC  TTTTTCGTCGCTTGGAGACT | 307 |
